# Selective Spin Dewetting for Perovskite Solar Modules Fabricated on Engineered Au/ITO Substrates

**DOI:** 10.3390/nano14050424

**Published:** 2024-02-26

**Authors:** Son Singh, Rahim Abdur, Md. Abdul Kuddus Sheikh, Bhabani Sankar Swain, Jindong Song, Jae-Hun Kim, Ho-Seok Nam, Sung-Hyon Kim, Hyunseung Lee, Jaegab Lee

**Affiliations:** 1 School of Advanced Materials Engineering, Kookmin University, Seoul 02707, Republic of Korea; ssingh@kookmin.ac.kr (S.S.); jony143@kookmin.ac.kr (R.A.); abdulkuddus@kookmin.ac.kr (M.A.K.S.); bsswain@kookmin.ac.kr (B.S.S.); hsnam@kookmin.ac.kr (H.-S.N.); 2 Center for Opto-Electronic Materials and Devices, Korea Institute of Science and Technology (KIST), Seoul 02792, Republic of Korea; 3 Department of Fashion Design, Kookmin University, Seoul 02707, Republic of Korea; kim_sunghyon@kookmin.ac.kr; 4 Department of Fashion Industry, Incheon National University, Incheon 22012, Republic of Korea

**Keywords:** perovskite mini-module, self-assembled monolayers (SAMs), hexadecanethiol (HDT), selective deposition, dewetting, connection electrode

## Abstract

We introduce a novel method for fabricating perovskite solar modules using selective spin-coating on various Au/ITO patterned substrates. These patterns were engineered for two purposes: (1) to enhance selectivity of monolayers primarily self-assembling on the Au electrode, and (2) to enable seamless interconnection between cells through direct contact of the top electrode and the hydrophobic Au connection electrode. Utilizing SAMs-treated Au/ITO, we achieved sequential selective deposition of the electron transport layer (ETL) and the perovskite layer on the hydrophilic amino-terminated ITO, while the hole transport layer (HTL) was deposited on the hydrophobic CH_3_-terminated Au connection electrodes. Importantly, our approach had a negligible impact on the series resistance of the solar cells, as evidenced by the measured specific contact resistivity of the multilayers. A significant outcome was the production of a six-cell series-connected solar module with a notable average PCE of 8.32%, providing a viable alternative to the conventional laser scribing technique.

## 1. Introduction

Hybrid organic–inorganic perovskites are of great interest owing to their outstanding optoelectronic properties [1,2,3,4,5] and easy and low-cost manufacturing processes [6,7]. These advantages pave the way for cost-effective and highly efficient solar cells. Consequently, efforts are underway to upscale perovskite solar cells into larger solar modules for market deployment [8,9,10,11,12]. Such modules can be constructed by dividing individual cells and integrating them via interconnections.

Traditionally, laser scribing has been preferred for creating thin-film solar modules [13,14,15,16,17,18,19]. This technique offers layer-by-layer precision in material removal using different laser wavelengths, facilitating detailed patterning of the modules with impressive resolution, processing speed, and selectivity. However, it is not flawless [20]. Laser damage [21,22], poor selectivity for the underlying layer, and metal particle contamination can lead to shunting [23,24,25,26]. Another shortcoming is the reliance on expensive equipment.

Selective patterning of solar cells can be an emerging alternative [27,28,29,30,31,32,33]. This method has the potential to address the challenges of laser processing. It offers a simplified, cost-effective means of producing modules with a high aperture ratio and is compatible with flexible manufacturing techniques.

A promising possibility in this field is the use of self-assembled monolayers (SAMs). SAMs are two-dimensional, ordered molecular arrangements on substrate surfaces. They possess the unique ability to modify surface properties based on their terminal groups. These groups determine the surface energy and functionality of the SAMs, making them either hydrophobic or hydrophilic [33,34,35,36,37]. This feature can be exploited for selective solvent wetting or dewetting and can also affect the interaction of the solute with the surface [38]. By leveraging these traits, the selective deposition of specific materials on predefined areas can be achieved, enabling precise solar module patterning.

In this research, we introduce a novel approach for producing perovskite solar minimodules, leveraging a selective patterning technique. This process utilizes two distinct self-assembled monolayers (SAMs) in combination with a spin-coating method. We employ a specially designed heterogeneous Au/ITO patterned substrate, which incorporates gold (Au) electrodes, termed connection electrodes, along the perimeter of the patterned indium tin oxide (ITO). This strategic arrangement facilitates the targeted deposition of SAMs: hydrophobic hexadecanethiol (HDT) on the Au electrodes and hydrophilic (3-Aminopropyl)triethoxysilane (APTES) on the ITO surfaces. Such configuration ensures the selective formation of SnO_2_ nanocrystals (NCs) and perovskite materials on ITO areas, enabling efficient interconnections among cell units without the drawbacks of laser scribing, such as metal particle contamination, high-temperature melting, and compromised selectivity. Our approach thus presents a non-damaging alternative that not only mitigates these issues but also validates the effectiveness of SAM technology in obtaining the precise patterning and seamless integration of perovskite solar cell (PSC) units, marking a significant leap in solar cell module fabrication.

Expanding on the system-level fabrication process for PSC modules, this study meticulously examines several pivotal factors impacting module performance, including the surface properties of SAMs, their selectivity, and the contact resistance. The SAMs’ formation was assessed using X-ray photoelectron spectroscopy (XPS), and the dynamic wetting and dewetting behaviors influenced by processing precursors were gauged through contact angle measurements.

A notable achievement of our study is the attainment of high pattern resolution in the sequential selective deposition of active layers, such as SnO_2_ NCs and perovskite, achieving approximately 100 µm-wide Au lines. This refinement significantly enhances the active aperture ratio of the modules. Moreover, we accomplished the seamless integration of interconnections between isolated cells through the direct contact of the top electrode on the hole transport layer (HTL) with the HDT-coated Au connection electrode, culminating in a multilayered contact structure (Au/HTL/HDT SAMs/Au). The efficacy of this intricate contact arrangement was corroborated using transmission line method (TLM) patterns, confirming the negligible resistance contribution from both the SAM and HTL layers.

The successful fabrication and electrical performance evaluation of six-subcell series-connected solar modules, using this methodology, demonstrate its potential for scaling up to larger modules. This advancement contributes to the ongoing development of PSC module technology, offering new possibilities for solar energy applications.

## 2. Material and Methods

### 2.1. Materials

All chemicals and materials were used as received, without further purification. Patterned ITO glass (10 Ω/sq) substrates were purchased from Freemteck (Seoul, Republic of Korea). Lead iodide (PbI_2_, 99.999% trace metal basis) was received from TCI (Tokyo, Japan). Methylammonium iodide (MAI, crystal) and 2,2′,7,7′-Tetrakis[N,N-di(4-methoxyphenyl)amino]-9,9′-spirobifluorene (Spiro-OMeTAD, 99%) were purchased from NCT Inc. (Seoul, Republic of Korea). Tin (IV) oxide (SnO_2_, 15% in H_2_O colloidal dispersion) was obtained from Alfa Aesar (Seoul, Republic of Korea). The chemicals, including hexadecanethiol (HDT), 3-aminopropyltriethoxysilane (APTES, 99.999%), methylamine hydrochloride (MACl, 98%), Li-bis(trifluoromethanesulfonyl)imide (Li-salt, 99.95%), dimethylsulfoxide (DMSO, 99.8%), N,N-dimethylformamide (DMF, 99.8%), chlorobenzene (anhydrous, 99.8%), 4-tert-butyl pyridine (TBP, 98%), acetonitrile (99.8%), 2-propanol, acetone, and ethanol were purchased from Sigma–Aldrich (Seoul, Republic of Korea).

### 2.2. Fabrication of Perovskite Minimodule

**Figure 1:**
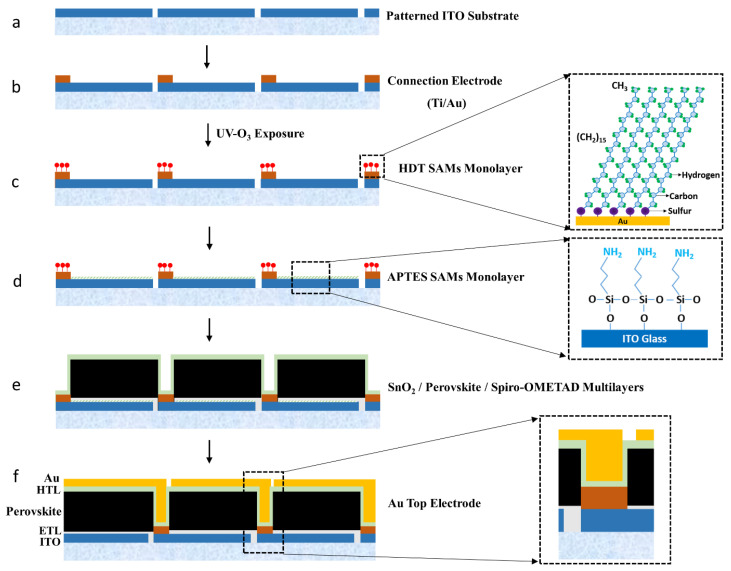
The schematic pathway for the selective deposition of active layers on patterned ITO cells employed to make a perovskite mini-module: (**a**) a patterned ITO glass substrate; (**b**) a heterogeneous (Au/ITO) structure fabricated by evaporating a Ti/Au bilayer connection electrode on the edge of each patterned ITO cell through a shadow mask; (**c**) HDT monolayers selectively assembled on UV-exposed Au connection electrode and the structure of HDT monolayers; (**d**) APTES monolayers selectively assembled on ITO surface and the structure of APTES monolayers; (**e**) selective deposition of the SnO_2_ NCs ETL and perovskite layer, followed by the non-selective deposition of the spiro-OMeTAD HTL; (**f**) thermally evaporated top electrode through the shadow mask, which was in contact with the connection electrode, leading to the multilayered contact structure, as shown in the inset of (**f**).

Figure 1 shows a schematic pathway for the selective deposition of active layers on patterned ITO cells to make a perovskite minimodule.

The patterned ITO glass substrate is a starting substrate, which was fabricated by wet etching using a photoresist (PR) mask produced through conventional photolithography (Figure 1a). The patterned ITO was cleaned ultrasonically in acetone, isopropyl alcohol, and deionized (DI) water for 10 min each and then dried with N_2_ gas. An E-beam evaporator under high vacuum (1 × 10^−6^ torr) was used to deposit the connection electrode consisting of Ti (100 nm)/Au (100 nm) through a shadow mask on the edge of each patterned ITO cell, making a heterogeneous (Au/ITO) structure (Figure 1b). The Au/ITO patterned substrate was then exposed to ultraviolet (UV)-ozone treatment for 30 min to activate the ITO surface. The HDT monolayers were then selectively assembled on the Au connection electrode of the cells by immersing the patterned glass in a solution of HDT (0.5 mM) in ethanol for 60 min at room temperature in an Ar-filled glove box, followed by rinsing with pure anhydrous ethanol and drying in N_2_ flow (Figure 1c). Next, the glass substrate was dipped in a solution of APTES (0.1 µL/mL) in pure anhydrous ethanol for 1 h at room temperature to attach APTES to an ITO surface (Figure 1d). The insets of Figure 1c,d show the structures of each SAM. Subsequently, the SnO_2_ NCs ETL and perovskite layers, dewetted from the hydrophobic connection electrodes, were selectively and sequentially spin-coated on a previously prepared ITO, followed by the non-selective deposition of a spiro-OMeTAD HTL on the substrate (Figure 1e). The conditions of the ETL and perovskite layer deposition were described in the previous work [38]. Finally, the top Au electrode (100 nm thick) was deposited using thermal evaporation under a high vacuum (1 × 10^−6^ torr) through a shadow mask to electrically connect the isolated cells, thus creating perovskite-based mini-modules (Figure 1f). The inset of Figure 1f shows a multilayered contact structure formed on the connection electrode for the integrated connection between cells.

### 2.3. Characterizations

The contact angles were measured using a Surface Electro Optics, SEO (Model: Phoenix 300) instrument at room temperature and in relative humidity of 30–35%. The DI water (18 MΩ.cm) and other solutions were dropped onto the sample surface using a syringe needle, and the droplet image was immediately recorded. The surface morphology was characterized using field-emission scanning electron microscopy (FESEM, JEOL-7610F, Tokyo, Japan). The X-ray photoelectron microscopy (XPS) data were analyzed utilizing a commercial system (K-alpha, Thermo VG, East Grinstead, UK) equipped with a focused monochromatic Al Kα source (h: 1486.6 eV) and a beam spot size 400 µm in diameter. The survey scans were controlled using a passing energy of 200 eV with a step size of 1 eV under an ultrahigh vacuum of 3.6 × 10^−9^ torr. The contact resistance of transmission line method (TLM) patterns was measured using a semiconductor parameter analyzer (HP4155C). The sheet resistances were measured using a four-point probe (CMT-series Changmin, Co., Ltd., Changwon City, Republic of Korea). The performance of the perovskite solar cell and mini-module was evaluated using a solar simulator (Newport Oriel Solar Simulator, Irvine, CA, USA) under AM 1.5G (100 mAcm^−2^) illumination. A standard Si solar cell (VLSI Standard, Newport Oriel, Irvine, CA, USA)) was employed to calibrate the intensity of illuminated light.

## 3. Results and Discussion

### 3.1. XPS Analysis of SAMs Assembled on UV-Exposed Substrates

The design and selection of self-assembled monolayers (SAMs) for surface modification require careful consideration of the interaction between the head group and the substrate, as well as the SAMs’ functionalization to achieve surface properties tailored to specific applications [34].

For enhancing the dewetting property of Au electrodes, HDT was chosen to create hydrophobic surfaces suitable for dewetting applications. This selection was based on the strong affinity of HDT’s thiol head group for the gold surface and the hydrophobic nature conferred by its lengthy hydrocarbon chains, which enhances dewetting. In addition, gold substrates are chosen for their superior ability to support uniform and durable SAMs with enhanced resistance to oxidation compared to other noble metals such as silver (Ag) and copper (Cu), thus underlining the rationale for our material selection in light of both performance and long-term cost-effectiveness considerations.

In contrast, for applications requiring a reactive surface, such as the attachment of SnO_2_ nanocrystals (NCs) to ITO substrates, APTES was selected. APTES creates a reactive amine-functionalized surface on ITO due to the silane group’s reaction with the hydroxyl groups on the ITO surface, forming a stable, covalently bonded siloxane layer. This functionalization facilitates the subsequent attachment of SnO_2_ NCs [38].

To optimize surface modification, two specific SAMs were utilized: the hydrophobic HDT-Au and the hydrophilic, reactive APTES-ITO. The ITO surface was activated using UV-ozone treatment prior to SAM assembly, improving the assembly of APTES. However, this treatment led to the oxidation of the Au surface. To assess the UV exposure effects on Au and its influence on HDT assembly and hydrophobicity, we conducted an XPS analysis.

**Figure 2:**
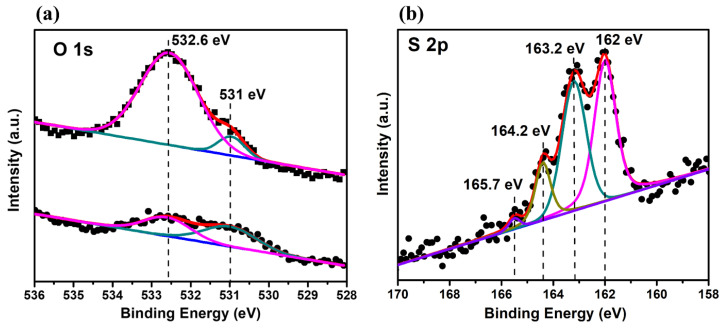
(**a**) The XPS spectrum of the UV-exposed Au (upper curve) and one post-ethanol dip at 25 °C for 20 min (lower curve) and (**b**) the S 2p XPS spectra of HDT SAMs on UV/ozone-treated gold.

Figure 2a presents the XPS spectrum of the UV-exposed Au (upper curve) and one post-ethanol dip at 25 °C for 20 min (lower curve). The O 1s data from the upper spectrum features two peaks at 531 and 532.6 eV, indicating hydroxide Au(OH)₃ and either adsorbed water or an oxyhydroxide AuOOH (Au₂O₃H₂O), respectively [39,40]. These curves fit well with minor differences in binding energies and peak gaps, suggesting an oxidic adlayer primarily of AuOOH and some Au(OH)₃ after UV/ozone exposure. Conversely, the O 1s data from the ethanol-treated UV-exposed Au (lower spectrum) reveal a significant decrease in AuOOH spectrum intensity, while the Au(OH)₃ intensity remains similar. This implies that ethanol treatment effectively reduces the amount of oxyhydroxide by retaining a slight mixture of AuOOH and Au(OH)₃ on the gold surface.

Additionally, after exposing the Au to UV, it was immersed in the HDT solution to produce an HDT-assembled Au surface. We then used XPS to determine the influence of the oxide layer on HDT assembly.

Figure 2b displays the S 2p XPS spectra of HDT SAMs on UV/ozone-treated gold. It comprises two doublets: a lower-intensity doublet at 164.2 eV (S 2p_3/2_) and 165.7 eV (S 2p_1/2_), indicating unbound sulfur atoms in HDT molecules and a stronger doublet at 162 eV (S 2p_3/2_) and 163.2 eV (S 2p_1/2_), denoting chemically bound sulfur on the gold surface [41,42,43,44,45]. These observations indicate that HDT monolayers self-assemble on UV/ozone-treated gold surfaces as a result of chemical interactions between sulfur and gold, despite the presence of a gold oxide layer. Possible reasons include ethanol rinsing reducing the AuOOH layer thickness and thiol molecules in the solution oxidizing on the Au(OH)₃ and AuOOH surfaces, thus forming Au–S bonds via hydration [40].

In the previous paper, we discussed the self-assembly of the APTES monolayer on ITO and its influence on subsequent SnO_2_ NCs ETL and perovskite active layer deposition. This monolayer on ITO covalently bonds with SnO_2_ NCs during spin-coating, leading to a uniform, pinhole-free SnO_2_ NC layer and promoting the consistent growth of the perovskite film [38].

### 3.2. Contact Angle Measurements of the Precursors on SAM-Treated Substrates

The phenomena of wetting and dewetting can be characterized by the contact angles of liquids forming on surfaces [46]. Typically, a contact angle ranging from 0° to 90° results in wetting, while angles exceeding 90° promote dewetting. Thus, the 90° mark serves as a general indicator of the transition from wetting to dewetting [47,48]. Additionally, the contact angle is affected by the solid substrate’s chemical composition and surface roughness, as well as the liquid’s chemical nature [49,50,51,52]. Taking this into account, we assessed the contact angle for all solutions used in our process on both HDT-assembled Au and APTES-assembled ITO.

**Figure 3:**
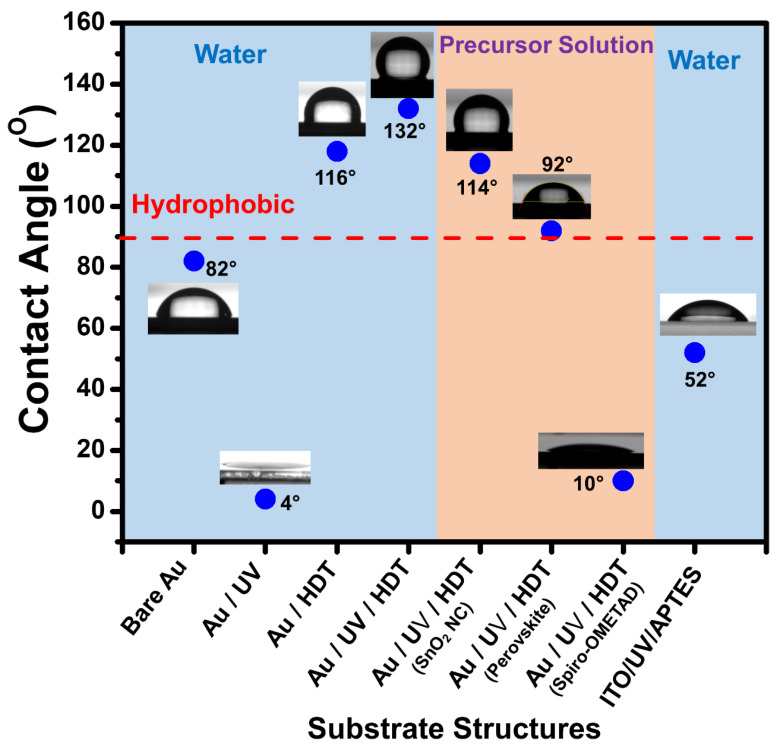
The water contact angles and the angles for precursor solutions, including the SnO_2_ precursor, perovskite precursor, and spiro-OMeTAD precursor, on bare Au, UV-treated Au, HDT-assembled bare gold, HDT-assembled UV-treated Au, and APTES-assembled ITO.

Figure 3 illustrates the water contact angles and the angles of precursor solutions, including the SnO_2_ precursor, perovskite precursor, and spiro-OMeTAD precursor, on both HDT-treated gold and APTES-assembled ITO. A water contact angle of 82° was recorded for bare Au. This angle rose to 116° on HDT-assembled bare Au and further increased to 132° on HDT-assembled UV-treated Au. Such a rise suggests that the HDT monolayer on the gold oxide, when retained on the Au surface, significantly enhances its water repellency compared to HDT on bare Au.

To understand the dewetting properties of our process’s precursors, we measured the contact angles for the SnO_2_ NC precursor, perovskite solution, and spiro-OMeTAD on HDT-assembled UV-exposed Au. The SnO_2_ NC and perovskite solutions exhibited contact angles of 114° and 92°, respectively. These values imply that the dewetting of the solutions on the HDT-assembled Au facilitated selective active layer patterns. On the other hand, the spiro-OMeTAD precursor had a notably low contact angle of 10°, indicating its wetting nature on the HDT surface. Finally, water’s contact angle on APTES-assembled ITO was 52°, signaling a hydrophilic character promoting the deposition of a uniform SnO_2_ layer through covalent bonding [38].

### 3.3. Specific Resistance Measurement of Multi-Layered Contact Structures

Given that spiro-OMeTAD was applied over the HDT-coated connection Au electrodes, it was essential to assess the impact of these patterning processes. We employed the TLM method [53] to evaluate three distinct structures: bare Au/Au; UV-treated Au/HDT SAMs/Au; and UV-treated Au/HDT SAMs/Spiro-OMeTAD/Au. Consequently, we derived four key parameters: specific contact resistivity ($\rho_{C}$), contact resistance (R_C_), sheet resistance (R_SH_), and transfer length (L_T_), using Equation (1).

**Figure 4:**
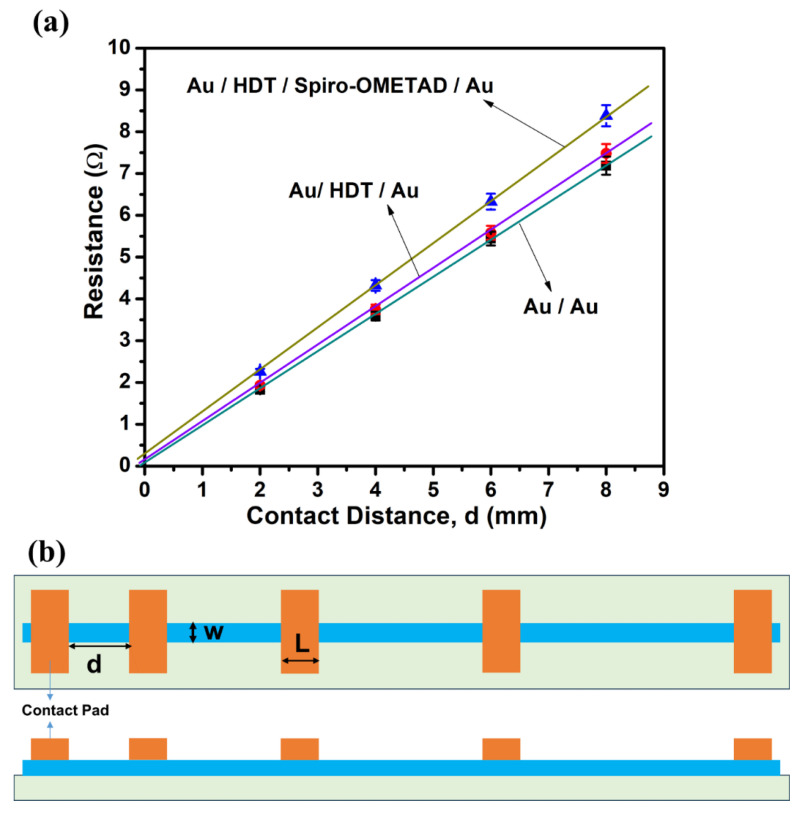
(**a**) TLM resistance measurements plotted against contact distance d for three different configurations including bare Au/Au, UV-treated Au/HDT SAMs/Au, and UV-treated Au/HDT SAMs/Spiro-OMeTAD/Au; (**b**) a schema of the TLM pattern, where L = 1000 µm and W = 500 µm.

Figure 4 illustrates (a) TLM resistance measurements plotted against contact distance for three distinct configurations including bare Au/Au, UV-treated Au/HDT SAMs/Au, and UV-treated Au/HDT SAMs/Spiro-OMeTAD/Au, as well as (b) a schematic of the TLM pattern, where L = 1000 µm, W = 500 µm, and d represents contact distance.

The total resistance (*R_T_*) between two contacts (having length L and width *W*) separated by distance d in TLM patterns can be described using the following [11]:

(1) $$R_{T}=2\;R_{C}+R_{SH}\;\cdot \;\left(\frac{d}{W}\right)$$

Here, *R_C_* stands for contact resistance, and *R_SH_* is the sheet resistance of the underlying Au.

From Equation (1), the sheet resistance of the Au substrate can be deduced from the TLM plot’s slope (equal to *R_SH_*/*W*). For the Au/HDT/Spiro-OMeTAD/Au configuration; it is approximately 0.51 ohm/square.

The transfer length, *L_T_*, is defined as the distance from a contact’s edge, where the current density value decreases to 1/e of its initial value. This can be expressed as [53,54]:

(2) $$L_{T}=R_{C}\;\cdot \;\left(\frac{W}{R_{SK}}\right)$$

where *R_SK_* is the sheet resistance of the Au substrate directly beneath the contact. Assuming no alloying or sintering at the metal-SAM layer interface, we consider *R_SK_* equal to the *R_SH_* of the Au substrate. Thus, *L_T_* is calculated as 98 µm (0.10 Ω × 500 µm/0.51 Ω/□).

Therefore, the specific contact resistivity ($\rho_{C}$) can be deduced:

(3) $$\rho_{C}=R_{C}\;\cdot W\;\cdot \;L_{T}$$

For Au–Au contact resistance, the result is negligible. In contrast, *R_C_* values for Au/HDT SAMs/Au and Au/HDT SAMs/Spiro-OMeTAD/Au are approximately 0.02 Ω and 0.10 Ω, respectively.

The SAMs’ contribution to specific contact resistivity is 2.17 × 10^−6^ Ω-cm^2^ (calculated as 0.02 Ω × 500 µm × 21.7 µm). Similarly, for Au/HDT SAMs/Spiro-OMeTAD/Au, it is 4.9 × 10^−5^ Ω-cm^2^ (0.10 Ω × 500 µm × 98 µm). Table 1 shows the extracted parameters for each contact structure.

It is noteworthy that this specific contact resistivity is below the 3–7 × 10^−4^ Ω-cm^2^ of Au/FTO (a conductive metal oxide) as reported in referenced studies [11,55], suggesting that the contact resistance of Au/HDT SAMs/Spiro-OMeTAD/Au is low enough to ensure optimal device performance.

### 3.4. Resolution in Selective Patterning from Au/ITO Line–Space Patterns

**Figure 5:**
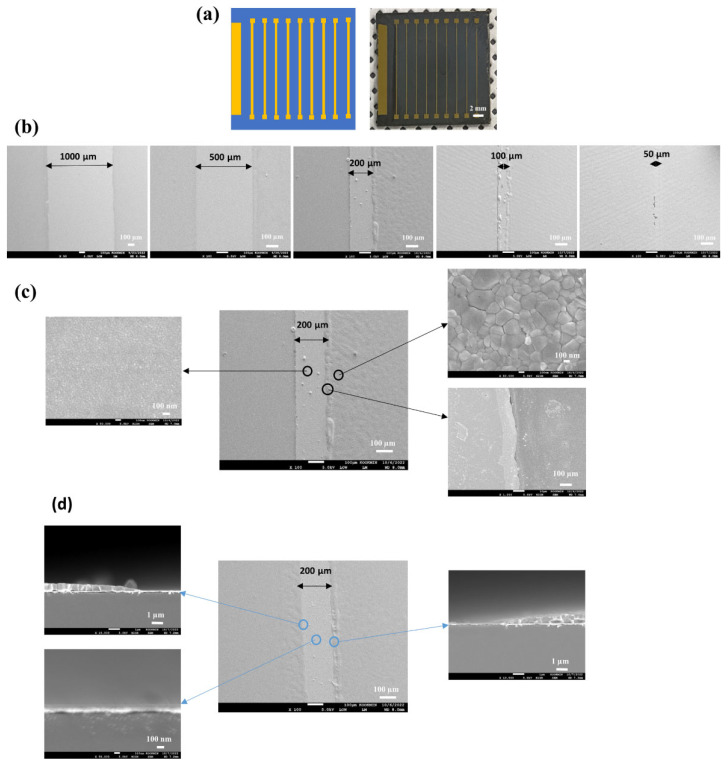
The sequential spin-coating of active layers such as SnO_2_ NCs and perovskite on Au/ITO line–space patterns: (**a**) A schemata of the heterogeneous Au/ITO line–space patterns and optical images showing 9 Au lines; (**b**) SEM images capturing the selective deposition of active layers on the Au/ITO patterns, with the line width varying from 50 µm to 1000 µm; (**c**) Close-up SEM images showing the selective perovskite deposition on ITO and a 200 µm-wide Au line devoid of SnO_2_ and perovskite with some coatings on the edges; (**d**) Cross-sectional SEM images revealing the deposition of SnO_2_ NCs and perovskite layers on ITO and the absence of deposition on a Au line. The insets in (**c**) demonstrate a clean Au surface (the left), perovskite microstructures (top on the right), and a wavy Au–perovskite interface (bottom of the right); The insets in (**d**) show layers of perovskite on ITO adjacent to a pristine Au/Ti line (top on the left), a clean Au/Ti line (bottom on the left), and perovskite deposition on ITO with an adjacent clean Au/Ti line.

Figure 5 illustrates the sequential spin-coating of active layers, namely, SnO_2_ NCs and perovskite, on mixed Au/ITO line–space patterns: (a) A schematic of the heterogeneous Au/ITO line–space patterns and optical images showing nine Au lines; (b) SEM images capturing the selective deposition of active layers on the Au/ITO patterns, varying line widths from 50 µm to 1000 µm; (c) Close-up SEM images demonstrate the selective perovskite deposition on ITO: it is evident that a 200 µm-wide Au line lacks deposition, although some coatings are present on the edges.

Sequential selective deposition pattern resolution was exhibited by successively spin-coating the SnO_2_ NCs and perovskite solutions onto the specialized Au/ITO patterns. Interestingly, lines with widths ranging from 1000 µm to 100 µm exhibited superior selective patterning; however, narrowing the width to 50 µm probably resulted in a loss of selectivity. It is worth noting that the right inset (bottom) in Figure 5c shows an undulating Au–perovskite interface near a 200 µm-wide Au line’s edge, spanning approximately 15 µm. This irregularity may result from the imperfect pinning of the hydrophilic solution on boundaries. For instance, a hydrophilic–hydrophobic interface could cause liquid to accumulate briefly before breaking free, resulting in a wavy interface. While several factors could influence this wavy growth, further investigations are required to comprehend and optimize the process.

Besides the intrusion of perovskite into the hydrophobic Au line, insets also draw attention to an Au surface devoid of perovskite, alongside the superior microstructure of the perovskite itself. The encroachment width of perovskite into the Au line fluctuates between 4 and 15 µm for Au lines with widths spanning from 100 µm to 1000 µm. Consequently, Au lines with a width equal to 50 µm likely lack selectivity due to perovskite’s lateral expansion from edge sites. These observations lead to the conclusion that reliable selective patterning is feasible up to an Au line width of 100 µm.

Regarding module efficiency, we examine a perovskite module that features a 1000 µm wide connection electrode. The module’s design delineates a dead area (Wd) of approximately 1900 µm and an active area (Wa) of 5200 µm. Given the total cell segment width (Wa + Wd) of 7100 µm, the active area fraction should be calculated using the formula Wa/(Wa + Wd), yielding a value of 0.73. This module represents the first instance of a perovskite module patterned by consecutively spin-coating active layers, achieving a remarkable aperture ratio.

Upon reducing the connection electrode width to 200 µm, our calculations show an increase in the active area ratio to 0.83. This is calculated as Wa/(Wa + Wd) = 5200 µm/(5200 µm + 1100 µm), a performance on par with that achieved through laser scribing methods. The improvement in the active area ratio to 0.96 is further facilitated by employing a 100 µm connection electrode in conjunction with advanced wet etching and shadow mask technologies for patterning the bottom electrode and the gold (Au) top electrode, respectively. The enhanced pattern resolution of approximately 50 µm reduces the total dead area width to 200 µm (100 µm for the connection electrode width; +50 µm for etching pattern resolution; +50 µm for shadow mask resolution), significantly elevating the efficiency of the module.

### 3.5. Electrical Performance of Perovskite Single Cells and Mini-Modules

We employed selective patterning techniques to produce six-segment series-connected perovskite solar modules. A single solar cell, measuring 0.09 cm^2^, was also fabricated using this selective process to serve as a performance benchmark for the larger modules.

An inset highlights a sample containing four individual cells, created through the selective method. Here, active layers were spin-coated on the APTES-coated active region of ITO and repelled from the HDT-coated Au electrodes situated on both the bottom and top ITO electrodes.

**Figure 6:**
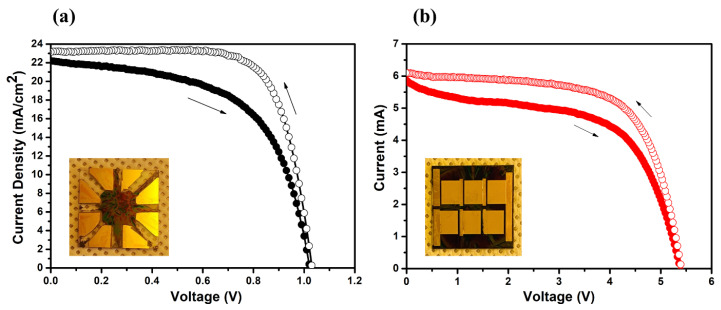
(**a**) The J–V curve of a single solar cell, which has an average PCE of 13.28%, a J_sc_ of 22.2 mA/cm^2^, a V_oc_ of 1.02 V, and an FF of 0.68 under forward bias; (**b**) the I–V curve of a solar module with six interconnected cells, yielding an average PCE of 8.93%, V_oc_ of 5.39 V, FF of 0.56, and J_sc_ of 2.94 mA/cm^2^: The solid and open circles represent forward scan and reverse scan, respectively. The insets display the optical images of a sample containing four single cells and a solar module with six interconnected cells fabricated based on the selective spin coating-based patterning method.

Figure 6a displays the J–V curve of a single solar cell, which has an average Power Conversion Efficiency (PCE) of 13.28%. It achieved a J_sc_ of 22.2 mA/cm^2^, a V_oc_ of 1.02 V, and a Fill Factor (FF) of 0.68 under forward bias (as summarized in Table 1). Analyzing the J–V curve, we extracted R_sh_ values near zero bias and an R_s_ value at a V_oc_ bias from the inverse of the J–V slope, respectively. These values are 500 Ω and 5.98 Ω, respectively. Such data suggest that our SAM-facilitated single cell exhibits superior selectivity over the HDT-coated Au electrode, combined with a high-performing solar cell that has notably low contact resistance and elevated shunt resistance.

In Figure 6b, the I–V curve represents the performance of a solar module composed of six interconnected cells. This configuration yielded an average PCE of 8.32%, V_oc_ of 5.39 V, FF of 0.56, and a J_sc_ of 2.76 mA/cm^2^. Furthermore, the module exhibited an R_sh_ of 600 Ω and an R_s_ of 12.31 Ω. (See Table 2). Comparatively, these results align closely with those of a single cell in terms of FF, shunt resistance, and selectivity, though the module’s series resistance shows a slight increase. Notably, the V_oc_ of 5.39 V falls short of the expected 6.0 V, likely due to uneven coatings on the active layer. Moreover, the observed discrepancy between forward and reverse bias scans can be mainly linked to the interactions at the SnO_2_–perovskite interface and the inconsistent deposition of active layers. To improve the performance of the module, future research will focus on refining the surface treatment of SnO_2_ nanocrystals (NCs). This step is essential for enhancing the interface between SnO_2_ NCs and the perovskite layer, leading to a more uniform deposition of the perovskite material. 

In summary, the module’s performance showcased a V_oc_ of 5.39 V, J_sc_ of 2.76 A/cm^2^, and FF of 0.56. This culminates in a PCE of 8.32% over an active area measuring 2.14 cm^2^. Although minor variations exist, the photovoltage remains closely aligned with that of individual cells. This consistency underscores the efficacy of our innovative spin process and the specialized connection electrode system, signifying our successful scale-up to larger modules.

## 4. Conclusions

In this paper, we introduced an innovative selective deposition-based patterning method for the fabricating of perovskite solar modules, effectively bypassing the need for traditional laser scribing techniques. Leveraging the unique characteristics of Au/ITO patterned cells, our approach utilized hydrophobic HDT-assembled Au as connection electrodes and hydrophilic APTES-functionalized ITO to optimize layer interactions during sequential spin dewetting.

Key findings and implications include:

Enhanced Selectivity: Our methodology ensured the selective deposition of active layers on Au/ITO patterns, achieving a patterning resolution of approximately 100 µm. This can be further optimized in future iterations.

Efficient Interface Interaction: UV exposure played a key role in improving the quality of the APTES monolayer on ITO, effectively promoting the interaction with SnO_2_ NCs ETL, and simultaneously forming a gold oxide layer on Au. This approach, confirmed using XPS, marked the hydrophobic properties of the assembled structure.

Low Specific Contact Resistivity: The integration of interconnection between the isolated cells was accomplished through the direct contact of the top electrode on the HTL coated on the HDT-Au connection electrode. As a result, the multilayered-contact structure consisting of Au/HDT/HTL/Au was obtained, and its specific contact resistivity measured using the TLM method was 4.9 × 10^−5^ Ω-cm^2^.

Promising Performance Metrics: The six-subcell series-connected solar modules, produced using our method, showed an average PCE of 8.32%, V_oc_ of 5.39 V, FF of 0.56, and J_sc_ of 2.76 mA/cm^2^.

This research emphasizes the potential of selective spin-coating-based patterning as a viable and scalable solution for large-scale perovskite solar module fabrication. Future work may focus on the further optimization of this technique and its possible applications in the solar industry.

## Figures and Tables

**Table 1:** The TLM parameters (L_T_, R_SH_, R_C_, ρ_C_) extracted for each contact structure.

Structure	L_T_ (µm)	R_SH_ (Ω/□)	R_C_ (Ω)	ρ_C_ (Ω-cm^2^)
UV-treated Au/HDT/Au	21.7	0.46	0.02	2.17 × 10^−6^
UV-treated Au/HDT/HTL/Au	98	0.51	0.10	4.9 × 10^−5^

**Table 2:** Performance parameters of the solar module and the single cell.

Sample		J_sc_ (mAcm^−2^)	V_oc_ (V)	FF	PCE (%)	R_sh_ (Ω)	R_s_ (Ω)
Single Cell	Forward	22.2	1.02	0.58	13.23	500	5.98
	Reverse	23.14	1.03	0.72	17.34	1890	4.66
Module	Forward	2.76	5.39	0.56	8.32	600	12.31
	Reverse	2.85	5.42	0.65	10.04	2381	8.24

## Data Availability

The data presented in this study are available upon request from the corresponding author.
